# Risk factors in Swedish young men for amyotrophic lateral sclerosis in adulthood

**DOI:** 10.1007/s00415-017-8719-1

**Published:** 2017-12-28

**Authors:** Maria Åberg, Jenny Nyberg, Josefina Robertson, Georg Kuhn, Linus Schiöler, Hans Nissbrandt, Margda Waern, Kjell Torén

**Affiliations:** 1 Department of Public Health and Community Medicine/Primary Health Care, Institute of Medicine, Sahlgrenska Academy, University of Gothenburg, Box 454, 405 30 Gothenburg, Sweden; 2 Centre for Brain Repair and Rehabilitation, Institute for Neuroscience and Physiology, Sahlgrenska Academy, University of Gothenburg, Gothenburg, Sweden; 3 Section of Occupational and Environmental Medicine, Department of Public Health and Community Medicine, Institute of Medicine, Sahlgrenska Academy, University of Gothenburg, Gothenburg, Sweden; 4 Department of Pharmacology, Institute of Neuroscience and Physiology, Sahlgrenska Academy, University of Gothenburg, Gothenburg, Sweden; 5 Department of Psychiatry and Neurochemistry, Institute of Neuroscience and Physiology, Sahlgrenska Academy, University of Gothenburg, Gothenburg, Sweden

**Keywords:** Amyotrophic lateral sclerosis (ALS), Motor neuron disease, Young men, BMI, Muscle strength, Erythrocyte volume fraction

## Abstract

Recent research suggests that the incidence of amyotrophic lateral sclerosis (ALS) may be on the rise. Since ALS becomes predominant in later life, most studies on causal factors are conducted in middle-aged or older populations where potentially important influences from early life can usually not be adequately captured. We aimed to investigate predictors in young Swedish men for ALS in adulthood. Therefore, we performed a prospective cohort study of young men (aged 16–25, *n* = 1,819,817) who enlisted 1968–2005 and took part in comprehensive conscription examinations. Incident cases of ALS (*n* = 526) during up to 46 years of follow-up were identified in the National Hospital Register and Swedish Cause of Death Register. Those who developed ALS had lower BMI (body mass index) at conscription than their peers (*p* = 0.03). The risk of ALS during follow-up was calculated with Cox proportional hazards models. No associations were found with physical fitness, erythrocyte sedimentation rate, or non-psychotic mental disorders. Low overall muscle strength compared to high overall muscle strength [hazard ratio (HR) 1.36; 95% confidence interval (CI) 1.01–1.83] and low BMI (a one-unit increase HR 0.96; 95% CI 0.93–0.99) and lower erythrocyte volume fraction (a one-unit increase HR 0.96; 95% CI 0.92–0.998) were the statistically significant predictors for ALS in adjusted models. These findings provide novel epidemiologic evidence of a prospective association between low overall muscle strength and erythrocyte volume fraction in young men and ALS risk.

## Introduction

Among the rare adult-onset motor neuron diseases, amyotrophic lateral sclerosis (ALS) is the most common. It is typically fatal within 2–5 years of symptom onset [1]. However, patients with early onset ALS commonly demonstrate a relatively longer survival [2]. The incidence of ALS is largely uniform across most parts of the world, but an increasing ALS incidence in recent years has been demonstrated both internationally [3] and in Sweden [4]. Although recent genetic studies have substantially improved our understanding of the causes of ALS, especially familial ALS [5], an important role of non-genetic factors in ALS is recognized. Elevated risk of ALS has been associated with premorbid smoking [6], low body mass index [7, 8], and high level of physical fitness [8, 9], whereas intake of antioxidants [10] and alcohol [11] seems to be protective. Regarding physical activity, the epidemiological evidence of associations with ALS is inconsistent [12–15]. One recent prospective cohort study demonstrated a weak inverse relationship between physical activity and ALS [16]. Another showed increased risk of ALS with higher physical activity [17]. A previous study based on the Swedish conscription register reported no association between muscle strength at age 18 and death in ALS [9]. However, those results were based on a limited number of cases, i.e., 85 ALS deaths and overall muscle strength were not studied. Furthermore, there is evidence for associations with occupational [18] and environmental exposures (reviewed in [19]), as well as different medical conditions [20, 21] and increased risk of ALS. Signs of neuroinflammation are found in a variety of diseases of the CNS including ALS [22], but it is unknown whether systemic inflammation precedes the onset of ALS.

A large record linkage study demonstrated a prospective association between mental disorders and a first diagnosis of ALS within the following year [23]. In that same study, a diagnosis of depression was associated with a first record of ALS after more than 5 years. The latter finding has raised the possibility that the development of depression in middle age may be an early marker for neurodegeneration. It has, to our knowledge, not been investigated if non-psychotic mental disorders occurring earlier in life may be associated with future ALS.

Our aim was to investigate potential predictors in young adults for ALS in adulthood. We, therefore, performed a prospective cohort study of Swedish young men enlisting for compulsory military service. These men were followed up to 46 years in national registers, resulting in over 500 ALS cases.

## Methods

### Population

**Fig. 1:**
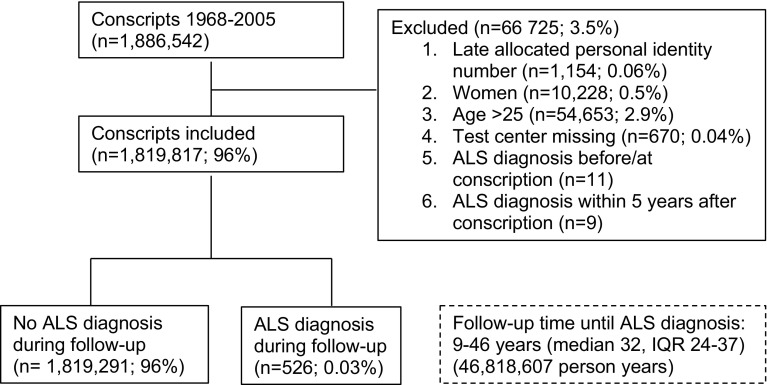
Flowchart illustrating the inclusion/exclusion, number of ALS diagnoses, and follow-up time of the Swedish male conscript study population, Sweden, 1968–2014, based on recommendations in Strengthening the Reporting of Observational Studies in Epidemiology (STROBE) [53]. *ALS* amyotrophic lateral sclerosis, *IQR* interquartile range

All Swedes have a unique personal identity number making linkage between different registers possible. Using this number, data from the Swedish Military Service Conscription Register were linked to the Swedish Hospital Discharge and death registries. The Swedish Hospital Discharge Register includes both inpatient (started 1986 and is complete since 1987) and outpatient visits (from 2001). A cohort of young individuals aged 16–25 (mean age 18.3, SD 0.7) who enlisted for military service between 1968 and 2005 (i.e., born between 1950 and 1987) was extracted from the conscription register (*n* = 1,886,542). At that time, all Swedish males had to enlist according to Swedish law. Exceptions were made regarding those who were imprisoned or had severe chronic somatic or mental conditions or functional disabilities documented by a medical certificate (approximately 2–3% annually). Exclusion criteria were: late allocated personal identity number (i.e., personal identity number reallocated from deceased person), female sex, ALS diagnosis before or at conscription, and missing data regarding conscription test center (Fig. 1). To further reduce baseline misclassification, we excluded incident ALS cases registered within 5 years after conscription (*n* = 9). The remaining 1,819,817 subjects were included in the present study (Fig. 1).

### Conscription register data

The enlisted men were examined over 2 days by psychologists and physicians at any of six conscription centers, Southern, Western, Eastern, Central (called Bergslagen), Northern lower, and Northern upper. The standardized procedure included measurement of weight and height.

#### Cardiovascular fitness test

Cardiovascular fitness was evaluated by a cycle ergometric test. During exercise, the work rate was successively increased until limited by exhaustion. The final work rate (*W*_max_) was recorded and divided by body weight. The resulting values (*W*_max_/kg) were converted into stanine scores that served as a measure of fitness. There were 394,633 men with only estimated values; these were not included in the analyses involving fitness. The test has previously been described in detail [9, 24].

#### Isometric muscle strength

Isometric muscle strength was measured by trained military personal according to methods developed by Törnvall [25]. Apparatus was calibrated on a daily basis. The strength of each muscle group was tested three times [26]. Hand grip strength of the strongest hand was measured in Newtons (N) in the standing position using a dynamometer devise with the upper arm being held vertically with the elbow flexed at 90°. Strength of right elbow flexion (N) was determined in the sitting position with the elbow held at 90° and the forearm held vertically. The strength of the right knee extension (N) was measured in the sitting position with the tested leg hanging vertically. The dynamometer was fastened at the level of the lateral malleolus.

#### Overall muscle strength

Weighted values for isometric muscle strength were measured by knee extension (weighted 1.3×), elbow flexion (weighted 0.8×), and hand grip (weighted 1.7×) [26]. They were integrated into an overall estimate in kilopond (until April 1st 1979) or Newton (after April 1st 1979), and divided into stanines. Test results were standardized using data from the previous years and converted to a 9-point stanine scale (low to high) to assure long-term stability of the data sets. These conversions have been performed for over 30 years by our collaborators at the National Service Administration in Sweden. We required at least one (of three) isometric muscle strength measurement for a conscript to get an overall estimate. Some had estimated muscle strength values (*n* = 43,509); these were excluded from the analyses involving muscle strength.

#### Blood samples

Venous samples were taken for analysis of the erythrocyte sedimentation rate (ESR) and the erythrocyte volume fraction (EVF, also known as hematocrit). The ESR, an indirect measure of inflammation, is defined as the distance that a column of anticoagulated blood falls in 1 h. It reflects a systemic response to inflammation, and is related primarily to fibrinogen and immunoglobulin concentrations [27]. The EVF is defined as the ratio of the volume occupied by red blood cells relative to the volume of whole blood [28]. ESR analyses were performed by the Westergren method, which is considered the reference method [29]. As the number of red blood cells influences the ESR, the EVF was used as a covariate in the Cox regression model involving ESR. The EVF analyses were performed by the microhematocrit method and were consistent with the National Committee for Clinical Laboratory Standards. ESR and EVF data were available for conscription years 1968–1984.

#### Psychiatric diagnosis

Psychiatric disorders were obtained from the Swedish Military Service Conscription Register. During enlistment, a psychologist evaluated psychiatric symptoms during a structured interview. Enlisted men with such symptoms were referred to a physician. First event of non-psychotic psychiatric disorders was diagnosed according to the *International Classification of Diseases* (*ICD*). Diagnostic codes for depressive disorders, neurotic/adjustment disorders, and personality disorders before or at conscription (Table 1) were pooled together in the model as non-psychotic disorders [30]. The distribution with each edition of ICD was 47,175; 13,085; 5,403 (ICD-8; ICD-9; ICD-10). In that model, individuals with hospital admissions for psychotic disorders including bipolar disorders (ICD codes: 296.1, 296.3, 298.1; 296.0, 296.2-5, 298.1; F30–31), schizophrenia (ICD codes: 295; 295; F20–21, F25) and other non-affective psychoses (ICD codes: 297.0-9, 298.2-3, 298.9; 297, 298.2-4, 298.8-9; F22–24, F28–29) with onset at any time were excluded (*n* = 35,870) to avoid misclassification of conscripts with prodromal episodes. In addition, since alcohol intake has been suggested protective, those with alcohol-related disorders and other substance use disorders at conscription (ICD codes: 291, 294.3, 303, 304; 291, 292, 303, 304, 305.0-8; F10–19, *n* = 9304) were excluded from the Cox model involving non-psychotic psychiatric disorders.

**Table 1:** Diagnostic categories and ICD codes for mental disorders, Sweden, 1968–2014

Diagnoses	No.	ICD codes (ICD-8; ICD-9; ICD-10)
Any non-psychotic mental disorders before/at conscription	65,663	
Depressive/neurotic disorders before/at conscription	56,413	296.0, 296.2, 298.0, 300.0-9, 305, 307; 298.0, 300.0-9, 306, 308-9, 311; F32–34, F38–48
Personality disorders before/at conscription	9832	301; 301; F60–69

Numbers of male conscripts with specified diagnostic categories and codes in accordance with the eighth, ninth, and tenth revisions of the ICD

*ICD* International Classification of Diseases

### Variables from other sources

#### Parental education

Information on parental education (highest level obtained up to year 2009) was taken from the longitudinal integration database for health insurance and labor market studies (Swedish acronym LISA, 80% coverage). For the purpose of this study, parental education (maternal and paternal education treated separately) was trichotomized as pre-high school (up to 9 years)/high school and < 2 years at university/≥ 2 years at university and postgraduate.

### Outcomes

#### Data sources

The universal healthcare system in Sweden offers low-cost outpatient and hospital care to all citizens. Swedish Hospital Discharge Register coverage gradually increased during the period 1968–1986, and is complete since 1987. Each patient is given a principal diagnosis and up to 30 secondary diagnoses at discharge. Diagnoses in hospital outpatient care are recorded from 2001. Deaths were identified by linkage with the Swedish Cause of Death Register, which is maintained at the National Board of Health and Welfare. This register is updated annually based on death certificate diagnoses and covers virtually all deaths since 1961.

#### ALS diagnoses

Cases were men who had a first time diagnosis of ALS (ICD 8 code 348.00, 348.10, and 348.21, ICD 9 code 335.2 and ICD 10 code G12.2) in the National Hospital Discharge Register or on their death certificate.

### Statistical analysis

The statistical calculations were performed with SAS version 9.4 (SAS Institute, Cary, NC, USA). The follow-up period started at the date of conscription (baseline) and subjects were followed until the time of: (1) first ALS hospitalization or hospital-based outpatient clinic contact, (2) death, (3) emigration, or (4) the end of follow-up, December 31, 2014 (minimum 9 years, maximum 46 years). For comparison of the population characteristics among men with and without ALS during follow-up, binary logistic regression was used for non-psychotic psychiatric diagnoses, multinomial logistic regression for conscription test center, ordinal logistic regression for ordered categories, and linear regression for continuous variables. Tests based on scaled Schoenfeld residuals were used to assess the proportional hazards assumption [31]. No significant violation was found for any of the variables in any of the models. Thus, there was no evidence for time-dependent associations, and Cox proportional hazards models were used to assess the influence of presumptive predictors and potential confounders for an ALS diagnosis.

The following presumptive independent predictors for ALS were treated as continuous variables: body mass index, *W*_max_, hand grip, elbow flexion, knee extension, ESR, and EVF. We examined deviations from linearity with restricted cubic splines, and found no significant deviations and no improvement in Akaike Information Criterion. Categorical variables were conscription test center, cardiovascular fitness, overall muscle strength, parental education, and non-psychotic psychiatric disorders. Cardiovascular fitness, overall muscle strength, and parental education were trichotomized as low, medium, and high. As temporal differences, as well as procedural differences among sites could result in bias, conscription year and test center were considered potential confounders and adjusted for in all regression models. Furthermore, age at conscription was adjusted for. Because of the large number of observations, the risk for type I errors is considered low.

For the exposure variables, overall muscle strength and cardiovascular fitness, respectively, which both had more than 10% missing values and over 5% difference between the proportions of missing values among ALS cases compared to non-cases, we performed analyses using multiple imputations by the fully conditional specification approach [32], using 20 imputations and 22 iterations. All variables included in any of the other analyses were included in the imputation model, with the exception of variables that were functions or transformations of other variables, such as grip strength per kg, where only the original variable was included. In addition, we included height, weight, blood pressure, resting heart rate, stress resilience, IQ and command suitability evaluated at the conscription and indicators for diabetes, death and emigration, and the estimated cumulative baseline hazard and event indicator for ALS as recommended in [33]. Convergence was assessed using plots of the mean and standard deviation and found satisfactory.

The Ethics Committee of the University of Gothenburg and Confidentiality Clearance at Statistics Sweden approved the study. Requirement for informed consent was waived for the current study, because it was a secondary analysis of existing data. The investigation conforms to the principles outlined in the Declaration of Helsinki.

## Results

Of the 1,819,817 conscripts in the present study, 526 (ICD-8, *n* = 10, ICD-9, *n* = 26 and ICD-10, *n* = 490) were diagnosed with ALS during up to 46 years of follow-up (Fig. 1). We used several registers for the analyses: hospital register, hospital outpatients register, and Cause of Death Register, which could be considered complete since 1987, 2001, and 1961, respectively. Almost all (*n* = 514) were identified by the Hospital Register. The cases ascertained from each source were: inpatient hospital only (*n* = 27), outpatient hospital only (*n* = 123), death only (*n* = 12), both inpatient and outpatient hospital (*n* = 98), both inpatient hospital and death (*n* = 45), both outpatient hospital and death (*n* = 13), all three sources (*n* = 208). The median age at first hospital inpatient/outpatient contact with an ALS diagnosis was 50.0 years. Many of the cases were identified by the hospital register at the very end of our observation period (e.g., 2010–2014); inpatient hospital (*n* = 52), outpatient hospital (*n* = 187), and both sources (*n* = 239), and 248 of all 514 (48%) identified by the Hospital Register were still alive in 2014. Half of the 12 deaths identified solely by the Cause of Death Register occurred prior to 2001, i.e., before outpatient data were included in the hospital register. The median age at death for cases captured solely in the Cause of Death Register was 43.5 years compared to 49.2 years for all deceased cases and 51.3 years for deceased cases only captured in the outpatient (but not inpatient) hospital register.

Table 2 shows baseline data for men with and without ALS diagnosis during follow-up. Numbers of missing values for non-cases/ALS cases were as follows (*p* values adjusted for calendar year): for BMI 150,882 (8.3%)/26 (4.9%) (*p* = 0.002), parental education 62,222 (3.5%)/37 (7.0%) (*p* = 0.72), overall muscle strength 201 084 (11%)/25 (4.8%) (*p* = 0.002), cardiovascular fitness 585,566 (32%)/97 (18.4%) (*p* = 0.47), ESR 57,089 (7.5%)/17 (3.8%) (*p* = 0.35), and EVF 59,447 (7.8%)/23 (5.2%) (*p* = 0.98). To investigate the impact of missing values on the results, we performed multiple imputation and reanalyzed overall muscle strength and cardiovascular fitness (see below). An association was seen with recruitment region. Baseline BMI was significantly lower in the ALS group. There was also a tendency towards a difference in overall muscle strength (*p* = 0.06), as shown by a smaller proportion of ALS subjects in the high muscle strength group. A tendency for lower erythrocyte volume fraction in the ALS group (*p* = 0.05) was also noted.

**Table 2:** Characteristics of enlisting men with and without ALS during follow-up, Sweden, 1968–2014

	No ALS (*n* = 1,819,291)	ALS (*n* = 526)	*p* value^a^
Age at baseline	18.3 (0.7)	18.5 (0.8)	0.052
Calender year, mean (SD)	1987 (10.3)	1977 (7.0)	< 0.001
Conscription region, no. (%)			0.001
South	414,493 (22.8)	134 (25.5)	
West	370,648 (20.4)	102 (19.4)	
East	434, 594 (23.9)	128 (24.3)	
Bergslagen	340,799 (18.7)	75 (14.3)	
North (lower)	176,987 (9.7)	37 (7.0)	
North (upper)	81,770 (4.5)	50 (9.5)	
BMI, mean (SD)	21.9 (3.0)	21.1 (2.5)	0.03
Parental education, no. (%)			0.41
Pre-high school (up to 9 years)	480,937 (27.4)	221 (45.2)	
High school and university (less than 2 years)	771,682 (43.9)	182 (37.2)	
University (2 or more years) and postgraduate	504,450 (28.7)	86 (17.6)	
Muscle strength^b^			0.06
Low, no. (%)	212,158 (13.1)	73 (14.6)	
Medium, no. (%)	914,695 (56.6)	315 (63.0)	
High, no. (%)	489,869 (30.3)	112 (22.4)	
Grip strength, N, mean (SD)	615.3 (98.1)	606.9 (102.8)	0.11
Grip strength per kg, N/kg, mean (SD)	8.9 (1.4)	9.1 (1.5)	0.35
Biceps strength, N, mean (SD)	387.3 (84.6)	376.3 (84.3)	0.53
Biceps strength per kg, N/kg, mean (SD)	5.6 (1.1)	5.6 (1.1)	0.19
Knee extension, N, mean (SD)	569.5 (118.1)	541.5 (113.1)	0.21
Knee extension per kg, N/kg, mean (SD)	8.2 (1.6)	8.1 (1.5)	0.56
Cardiovascular fitness^c^			0.56
Low, no. (%)	167,259 (13.6)	70 (16.3)	
Medium, no. (%)	724,134 (58.7)	238 (55.5)	
High, no. (%)	342,332 (27.7)	121 (28.2)	
*W*_max_, mean (SD)	280.5 (53.1)	256.9 (49.7)	0.90
*W*_max_/kg, mean (SD)	4.0 (0.7)	3.8 (0.7)	0.48
Erythrocyte sedimentation rate, mm/h, mean (SD)	3.3 (3.3)	3.5 (3.6)	0.32
Erythrocyte volume fraction, %, mean (SD)	46.4 (2.4)	46.2 (2.5)	0.05
Non-psychotic mental disorder,^d^ no. (%)	65,630 (3.7)	33 (6.4)	0.98

*ALS* amyotrophic lateral sclerosis, *BMI* body mass index, *W*_*max*_ maximal workload; *NS* non-significant, *mm* millimeter, *h* hour, *kg* kilogram, *N* newton, *SD* standard deviation

^a^ *p* values were adjusted for calendar year

^b^Performance was trichotomized as low (score 1–3), medium (score 4–6), and high (score 7–9)

^c^Performance was trichotomized as low (score 1–4), medium (score 5–7), and high (score 8–9)

^d^Before/at conscription

We evaluated conscription region, BMI, parental education, overall muscle strength (including each separate subparameter: grip strength, biceps strength, and knee extension), cardiovascular fitness, erythrocyte sedimentation rate, erythrocyte volume fraction, and mental disorder in the relation to the risk of developing ALS (Table 3). BMI, erythrocyte volume fraction, and overall muscle strength were the variables prospectively associated with ALS in analyses adjusted for age, conscription year, and conscription test center. A one-unit increase in BMI or erythrocyte volume fraction was associated with lower ALS risk. Low muscle strength at conscription was associated with a 1.4-fold increase for ALS later in life, compared to high muscle strength. Sensitivity analyses in which we repeated the analysis for muscle strength also including men with estimated scores yielded similar findings. Performing subanalyses for each muscle strength stanine score revealed that a significant risk increase was found for stanine 1 only, with unadjusted HR 4.3 (1.03–17.8) comparing stanine 1 with 9 (no differences were found for stanines 2–8, data not shown). The analysis using the imputed dataset showed adjusted HR 1.32 (0.98–1.77) for low muscle strength and adjusted HR 1.14 (0.91–1.41) for medium muscle strength (both compared to high overall muscle strength).

**Table 3:** Hazard ratio for ALS by adjusted (HR were adjusted for age, calendar year, and region except for analyses of conscription region which were adjusted for age and calender year only) risk factor, Sweden, 1968–2014

	HR	95% CI
Conscription region, no. (%)		
South^a^	0.66	0.48, 0.92
West^a^	0.59	0.42, 0.83
East^a^	0.62	0.44, 0.86
Bergslagen^a^	0.50	0.34, 0.70
North (lower)^a^	0.51	0.34, 0.79
BMI	0.96	0.93, 0.99
Parental education		
< 2 year university^b^	1.04	0.80, 1.34
> 2 year university^b^	0.99	0.77, 1.28
Muscle strength		
Low^c^	1.36	1.01, 1.83
Medium^c^	1.16	0.93, 1.44
Grip strength, N, per 100-unit	0.93	0.85, 1.02
Grip strength/kg	1.03	0.96, 1.09
Biceps strength, N, per 100-unit	0.97	0.87, 1.08
Biceps strength/kg	1.04	0.96, 1.12
Knee extension, N, per 100-unit	0.95	0.88, 1.03
Knee extension/kg	1.01	0.96, 1.07
Cardiovascular fitness		
Low^c^	1.07	0.79, 1.43
Medium^c^	1.07	0.86, 1.33
*W*_max_, per 50-unit	1.00	0.88, 1.13
*W*_max_/kg	1.07	0.89, 1.29
Erythrocyte sedimentation rate, mm/h	1.01	0.98, 1.03
Erythrocyte volume fraction, %, mean (SD)	0.96	0.92, 0.998
Non-psychotic mental disorder^d^	0.95	0.67, 1.35

*ALS* amyotrophic lateral sclerosis, *BMI* body mass index, *CI* confidence interval, *HR* hazard ratio, *W*_*max*_ maximal workload, *mm* millimeter, *h* hour, *kg* kilogram, *N* newton

^a^Reference category: north (upper)

^b^Reference category: pre-high school (up to 9 years)

^c^Reference category: high

^d^Reference category: no diagnosis of non-psychotic mental disorder before or at conscription

There was also an increased risk of ALS in the upper north recruitment region compared to the other regions (shown in Table 3 as a decreased risk of ALS in all other regions when the upper north region was reference category). We found no prospective relationship between cardiovascular fitness and ALS. The analysis using the imputed data set yielded similar associations with adjusted HR 1.12 (0.85–1.49) for low fitness and adjusted HR 1.07 (0.86–1.33) for medium fitness (both compared to high cardiovascular fitness).

A sensitivity analysis in which men with estimated *W*_max_ scores were included yielded similar findings (data not shown). Additional adjusting for BMI did not affect the results.

Non-psychotic mental disorders were not associated with ALS. A separate analysis of the association between alcohol/drug disorders (*n* = 9,304) was not meaningful due to lack of statistical power (only 2 ALS cases).

## Discussion

Low muscle strength, low BMI, and lower erythrocyte volume fraction in young men were associated with increased risk of ALS in this large male cohort. We did not find support for the previously reported association with physical fitness at conscription. No associations were found with parental education, erythrocyte sedimentation rate, or psychiatric disorders.

### Possible mechanisms

To our knowledge, this is the first study to demonstrate a prospective relationship with low overall muscle strength measured decades prior to a clinical diagnosis of ALS. It has recently been shown that respiratory muscle strength is a strong predictor for survival in ALS [34]. In line with Mattsson et al. [9], we found no association between each separate subparameter of muscle strength, i.e., grip strength, biceps strength, and knee extension and later ALS. As in the previous studies [7, 9], we found that low BMI was associated with future ALS. These findings suggest that the previously reported association between BMI and future ALS may actually reflect early muscular parameters.

It has been suggested that there is a risk phenotype for ALS characterized by relatively more type 1 muscle fibers (slow twitch fibers) [9]. Type 1 muscle fibers are the predominant muscle fiber type in endurance sports which may explain the higher incidence of ALS among professional athletes [19, 35]. The premorbid overall lower muscle strength seen in the present study could, perhaps, be explained by a smaller proportion of the larger type 2b fibers and in particular to the lesser force generating capacity of type 1 compared with type 2b fibers [36]. Muscle biopsies demonstrated that type 1 fibers are affected earlier and more severely in ALS [37]. There are so far no data on premorbid muscle fiber pathology in ALS.

Other plausible premorbid mechanisms may be motor nerve hyperexcitability [38], androgen insensitivity [39, 40], and inflammation [41, 42]. In the current study, we did not find evidence of an association between a systemic marker of inflammation (erythrocyte sedimentation rate) and future ALS. However, a significant association was observed for erythrocyte volume fraction (EVF), i.e., each percent increase was associated with a 4% decreased risk of later ALS. Although changes in EVF could predict disease progression in patients diagnosed with ALS [43], there are, to our knowledge, no previous reports indicating lower EVF as an early predictor of ALS. The present observation thus needs further investigation and it is far too early to speculate in mechanisms such as motor neuron and skeletal muscle oxygenation.

Somewhat surprisingly, the previously reported prospective association with high level of physical fitness in Swedish conscripts [8, 9] was not confirmed in our larger cohort. In addition to sample size, some other differences may help to explain the divergent results. The previous Swedish conscription study by Mattsson et al. only studied men born between 1951 and 1965 (based on deaths only, *n* = 85 cases), whereas we studied men born between 1950 and 1987 which means that the differences could be due to temporal trends. In our study, 42% of the ALS cases were still alive [9]. In addition, a recent Swedish conscription study by Longinetti et al. [8] showed that individuals with physical fitness above the highest tertile tended to have a higher risk of ALS before the age of 45 years, statistically significant associations only at age 41–43 years at ALS onset and only after adjustment for BMI and resting heart rate. Our cohort size is similar to that of the Longinetti et al. study, but our longer follow-up time yielded 87 additional ALS cases. The study of Longinetti et al. compares conscripts with high physical fitness (*W*_max_/kg: ≥ 4.25 W/kg) with conscripts with lower physical fitness in fitted flexible parametric models with attained age as the time scale. We found no evidence for time-dependent associations for cardiovascular fitness or *W*_max_/kg in any of the models.

As expected due to the higher incidence of ALS in the background population [44], men who enlisted in Northern Sweden were more likely to develop ALS than their peers who enlisted at other sites. The most plausible explanation for this is a genetic vulnerability. Familial aggregation and genetic risk factors have been estimated to explain 10–15% of all ALS cases [45]. One of the two major genetic contributions to ALS known to date is the C9ORF72 gene at chromosome 9 which is overrepresented in Northern Sweden [19]. In addition, persons living in northern Sweden might be differentially exposed to (unknown) environmental factors.

We could not detect any associations between non-psychotic mental disorder at conscription and later ALS. The previously demonstrated association with psychiatric disorders occurring more proximate in time [23] might suggest that mental disorders are prodromal symptoms of ALS-related neurodegeneration rather than predictors or risk factors per se.

### Strengths and limitations

Strengths of this study include the large sample size (> 1.8 million participants) and the prospective population-based design. The study population is largely representative of the male general population as only a small proportion (approximately 2–3% annually) of Swedish men were exempted from conscription prior to 2005. The follow-up was long, allowing for a considerably larger number of ALS cases compared to the previous studies employing the conscription register. The Swedish Hospital Discharge Register has a high accuracy for chronic diseases [46]. Both the Hospital Discharge Register [47] and the Swedish Cause of Death Register [9] have been shown to provide accurate ALS data.

The data retrieval of non-psychotic disorders from conscription data is a strength of the present study including psychologists and medical doctors for baseline measure of mental health at conscription. The general incidence is likely to be underestimated for non-psychotic disorders in the Hospital Discharge Register, since these disorders are mostly seen in primary care (where no national registers are available). This is less of a concern for estimates for psychotic disorders (diagnostic codes used for exclusion only to avoid misclassification of conscripts with prodromal episodes), as most persons with these disorders will come to the attention of psychiatric services at some point in time. However, psychotic disorders could be underestimated in this study, because outpatient hospital data, which are likely more sensitive than inpatient data, are only available for 2001–2014. We believe that it is unlikely that the associations we examined would be particularly sensitive to this lack of information which should be similar for individuals with and without an ALS diagnosis. Psychiatric diagnosis codes may change over time with each edition of ICD. The diagnostic validity of the schizophrenic disorders in the Swedish National Hospital Discharge Register has been shown to be high [48]. While not all mental diagnoses have been individually validated, the positive predictive values for most ICD diagnoses in the Swedish National Hospital Discharge Register are reported to be 85–95% [46].

There are further limitations to our study. For some exposure variables, for example, overall muscle strength and cardiovascular fitness, the amount of missing values could have biased the results. However, adjusting for the impact of missing values with multiple imputations showed similar associations, although it did not reach statistical significance for low muscle strength. The study population is homogeneous, with a majority of Caucasian males at the age of 18, which confines the generalizability to other populations, and to women. A draw back of the present study is that ALS cases may be under ascertained, since outpatient hospital data, which are likely more sensitive than inpatient data, are only available for 2001–2014. Before 1987, a case had to be diagnosed with ALS, die from it, and have ALS recorded on their death certificate to appear in the Cause of Death Register. There is a lag between diagnosis and death (probably 5–10% of ALS patients survive more than 10 years after diagnosis), so we may not have captured all ALS cases. In addition, a median age of 50.0 years for ALS cases is a little bit younger than what has typically been reported (i.e., 55 or 60 years) [1]. There are two likely explanations for this. First, men who signed for military service in the recent years were not old enough to develop ALS at the end of the observation period, and second, it has been shown that women have a later onset-age compared to men [49]. A median age at death of 43.5 years regarding the 12 cases captured solely in Cause of Death Register is about 20 years younger than what has typically been reported which was probably due to a selection of individuals with an early onset ALS. The median age at death was higher when all cases that died were included.

Our study design did not allow for the examination of youth metabolic factors that may be of importance for BMI and the development of ALS. Obesity and type 2 diabetes (not type 1) have been shown protective for ALS [21, 50]. Recent research shows that alterations in the carbohydrate, lipid, and apolipoprotein metabolisms are associated with ALS risk [51]. High glucose level was associated with a lower incidence, whereas high LDL-C/HDL-C and high apoB/apoA-I ratios were associated with a higher ALS incidence. Higher levels of LDL and apoB might affect the energy supply to the nervous system. Increased apoB has been associated with inflammation and oxidative stress—a common disease mechanism of ALS [52]. In addition, we were not able to study the development of certain exposures that may affect the risk of ALS such as smoking habits, diet, physical activity, exposure to toxins, and occupation during follow-up.

## Conclusion

In conclusion, we found that low overall muscle strength in young men was associated with increased risk of ALS. The previously reported prospective association with low BMI could in part be a reflection of low overall muscle mass. Additional explanations may include obesity, diabetes, and unhealthy lipid profiles. Our study provides an interesting lead for further ALS research to determine whether a premorbid neuromuscular constitution or pathogenesis may exist already in young adults.
